# A Parrot Story

**Published:** 1887-08-06

**Authors:** F. O. Morris

**Affiliations:** Rector of Nunburnholme, Yorkshire; author of "A History of British Birds," etc.


### INCIDENTS OF ANIMAL LIFE.

COLLATED BY THE REV. F. O. MORRIS, B.A., Rector of Nunburnholme, Yorkshire; author of "A History of British Birds," etc.

"He prayeth best, who loveth best
All things both great and small;
For the dear God who loveth us,
He made and loveth all."—COLERIDGE.

### A PARROT STORY.

A FEW years ago I was steaming down the Garonne, between Bordeaux and Pauillac, to stay with some English friends (who were also on board) at their château in Médoc. Every two or three miles down the river the vessel stopped to take on board or land passengers, when the customary short command, "Stop her!" was given (in English) by the skipper. After this had taken place some three or four times, and the steamer was well on her way to the next landing-place, a voice from amidships suddenly sang out, "Stop her!" The vessel was stopped accordingly, and the two seamen ran to the side to receive the expected passengers, but no passengers appeared; and the order was given to "Go ahead!" A few miles further on, "Stop her!" again resounded from the same quarter, and the vessel was again stopped, with the same futile result. This absurd scene was enacted several times during the passage, amid sundry maledictions from the captain and crew at being so often "made fools of"; till at last the true culprit was betrayed by an "eldritch laugh" from an old grey parrot, belonging to my English friends, whose cage had been placed close by the engine-room. His quick ear had caught the word of command, which he reproduced in the tantalising manner I have described; and the best of it was that the wretch seemed thoroughly to enjoy the confusion he had created, as if he understood all about it!

